# Improving Measurement Accuracy of Deep Hole Measurement Instruments through Perspective Transformation

**DOI:** 10.3390/s24103158

**Published:** 2024-05-16

**Authors:** Xiaowei Zhao, Huifu Du, Daguo Yu

**Affiliations:** 1 School of Mechanical Engineering, North University of China, Taiyuan 030051, China; b20220206@st.nuc.edu.cn (X.Z.); b20230211@st.nuc.edu.cn (H.D.); 2 Shanxi Deep Hole Processing Engineering Technology Research Center, Taiyuan 030051, China

**Keywords:** deep hole measurements, error compensation, perspective transform matrix, pixel drift

## Abstract

Deep hole measurement is a crucial step in both deep hole machining and deep hole maintenance. Single-camera vision presents promising prospects in deep hole measurement due to its simple structure and low-cost advantages. However, the measurement error caused by the heating of the imaging sensor makes it difficult to achieve the ideal measurement accuracy. To compensate for measurement errors induced by imaging sensor heating, this study proposes an error compensation method for laser and vision-based deep hole measurement instruments. This method predicts the pixel displacement of the entire field of view using the pixel displacement of fixed targets within the camera’s field of view and compensates for measurement errors through a perspective transformation. Theoretical analysis indicates that the perspective projection matrix changes due to the heating of the imaging sensor, which causes the thermally induced measurement error of the camera. By analyzing the displacement of the fixed target point, it is possible to monitor changes in the perspective projection matrix and thus compensate for camera measurement errors. In compensation experiments, using target displacement effectively predicts pixel drift in the pixel coordinate system. After compensation, the pixel error was suppressed from 1.99 pixels to 0.393 pixels. Repetitive measurement tests of the deep hole measurement instrument validate the practicality and reliability of compensating for thermal-induced errors using perspective transformation.

## 1. Introduction

The rapid development of deep hole components such as hydraulic cylinders, axles, and gun barrels relies heavily on the technological support from the fields of deep hole machining and measurement. The geometric precision during the processing of deep hole components and the internal wall damage during usage directly affect their performance [1,2,3]. Only by obtaining the internal parameters of deep hole components can various geometric quantities such as diameter [4,5], roundness and straightness [6], and internal wall damage [7,8,9] be evaluated. Currently, the measurement of deep hole components overly relies on manual methods, with lever-type diameter measuring instruments commonly used for dimensional measurements and endoscopes for damage assessment. These methods are inefficient and cannot be used for a quantitative analysis of measurement results. In recent years, the rapid development of optoelectronic technology has seen researchers employing techniques such as stable multi-color multi-ring lights [10], stripe lights [11], and point lasers [12] to project structured light onto hole walls and using cameras to capture images of structured light for deep hole inner wall measurement, thus advancing deep hole measurement technology.

However, in measurement technologies based on vision and structured light, the imaging quality of the camera determines the measurement accuracy. In recent years, attention has been drawn to deviations in camera imaging caused by temperature [13,14,15,16]. Adamczyk and Shi chao Zhou, among others, have analyzed the thermal behavior of cameras, finding that deformations caused by self-heating are unpredictable [17,18]. After implementing constraints on the camera’s degrees of freedom, they fitted a model correlating pixel drift with temperature, establishing a compensation model. This model, due to its numerous constraints, demands strict mounting methods for the camera and has limited applicability. Huafei Zhou used a static target to calibrate the thermal effects at different temperatures, fitting a first-order polynomial model for pixel displacement. He detailed the thermal-induced variability of polynomial parameters, creating a correlation model between these parameters and camera temperature [19]. This method of using different models for different temperatures proves more effective. Lei Xing proposed a dual-view system [20]. This system captures images of unknown targets and known mask images simultaneously, using changes in the known images to compensate for unknown variations, thereby achieving better compensation results. However, deformations in various components can cause shifts in the mask position, introducing new errors during the compensation process. Although some studies indicate that model compensation methods can reduce the impact of temperature on measurement results, Qifeng Yu discovered that thermal-induced pixel drift does not correspond directly with temperature. The coupling between ambient temperature and self-heating makes it challenging to establish an ideal compensation model for the pixel drift related to temperature values [21].

In summary, current compensation methods primarily focus on model-based compensation. However, pixel drift caused by camera heating is influenced by numerous factors, with multiple thermodynamic behaviors underlying the drift. Exploring the mechanism of this drift starting from temperature alone is insufficient for a comprehensive and detailed analysis of thermal behavior. In complex environments, where ambient temperatures are random, model compensation methods may fail. Currently, thermal-induced errors remain a significant source of error in visual measurement, urgently necessitating an effective compensation method to address temperature-induced inaccuracies.

In this paper, we introduce a deep hole inspection instrument based on circular laser and vision and propose a method of compensating for the thermal-induced pixel drift of the camera based on perspective transformation. In this method, we assume that regardless of temperature changes, the pixel drift between each pixel in the imaging sensor is correlated. Based on this assumption, we propose a method to predict the pixel drift of the entire imaging plane by using the perspective transformation matrix solved by the offset parameters of several known points, so as to compensate for the heat-induced pixel drift. The experimental results show that the camera thermal-induced pixel drift compensation method based on perspective transformation can effectively compensate for the visual measurement errors caused by camera pixel drift and effectively improve the measurement accuracy of deep hole measurement instruments.

## 2. Principle of Deep Hole Measuring Instrument and Compensation Methods

### 2.1. Measuring Instrument and Principle

**Figure 1:**
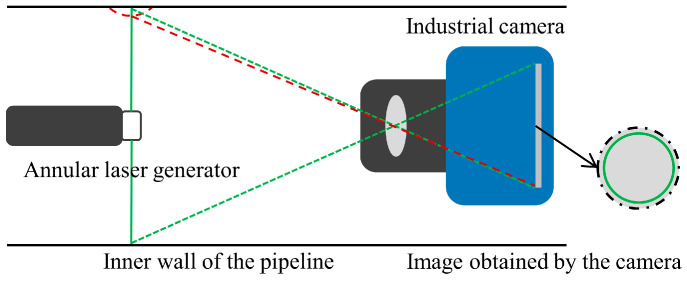
The principle of deep hole measurement.

A deep hole measurement method is proposed based on laser and monocular vision, and its measurement principle is shown in Figure 1. The ring laser projects a beam onto the hole wall, forming a laser ring. An industrial camera captures images of the laser ring along the axial direction of the deep hole. Changes in the edge of the inner hole cause the position of the laser ring to shift. By processing the collected laser ring images and extracting contours, the diameter information of the hole section can be obtained.

**Figure 2:**
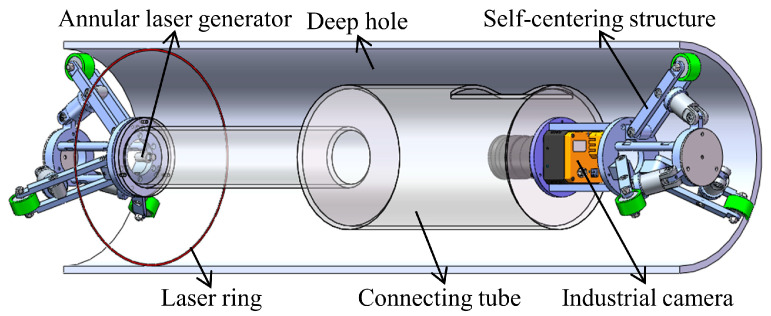
The structure of the measurement instrument.

Based on the above principle, Figure 2 illustrates the deep hole measurement instrument we have designed. During the measurement process, the wheel-type self-centering device closely contacts the inner wall of the deep hole and moves along the axis of the hole. The laser device is fixed on the self-centering device, and its beam forms a laser ring on the hole wall, generating structured light in a planar circular pattern. The CCD industrial camera, fixed on the self-centering device, continuously captures images of the laser ring on the inner wall of the deep hole. By identifying the sub-pixel contour and coordinates of the laser ring in each frame, the diameters of various cross-sections can be obtained.

**Figure 3:**
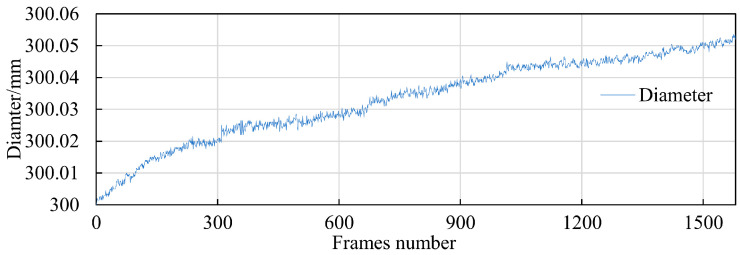
The diameter results measured by the deep hole measurement instrument.

The deep hole to be measured has a diameter of 300 mm, with a required measurement accuracy of ±0.03 mm. To verify the measurement accuracy of the instrument, we conducted a repeatability test using a 300 mm ring gauge, which has a dimensional accuracy of ±0.005 mm. The camera’s field of view is 320 mm × 320 mm, achieving a resolution of 0.0625 mm per pixel. In this setup, each pixel shift results in an error of 0.0625 mm. Theoretically, by using sub-pixel positioning, changes in measurement results should be controlled within 0.016 mm. During the test, the measurement instrument repeatedly measured the diameter of a fixed section of the ring gauge. The only variable in the entire measurement system was the temperature change caused by the camera’s self-heating. Figure 3 shows the results of the instrument’s repeatability test. The initial measurement was 300.0015 mm, and not only did it show small fluctuations, but it also displayed a significant increasing trend. After 1580 cycles of repeated measurements, the diameter measured increased by about 0.057 mm due to temperature changes. The presence of this error makes it difficult to meet the measurement requirements.

### 2.2. Theory of Compensation

**Figure 4:**
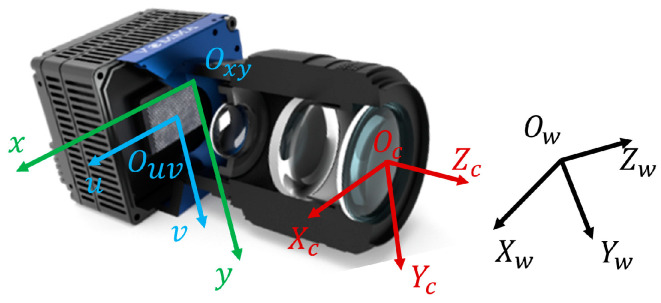
The schematic diagram of the coordinate system.

The process of visual imaging can be described by the transformation from the world coordinate system to the pixel coordinate system [22]. Figure 4 depicts four coordinate systems in visual imaging. The world coordinate system (Xw, Yw, Zw) serves as the reference for the object’s position, with its origin and orientation freely set for computational convenience. The camera coordinate system (Xc, Yc, Zc) has its origin at the optical center of the lens, with the Z-axis parallel to the optical axis of the lens. The transformation from the world coordinate system to the camera coordinate system involves rigid body transformation, including rotation and translation, used to describe the spatial position of the camera. The image coordinate system (x, y) is in units of meters or millimeters, established with the image center as the origin; the transformation from the camera coordinate system to the image coordinate system is a perspective projection transformation, representing a conversion from 3D to 2D. The pixel coordinate system (u, v) is in units of pixels, with its origin at the top-left corner of the image. The transformation from the image coordinate system to the pixel coordinate system involves only the conversion from mm to pixels, comprising translation and scaling.

The thermal-induced error of the camera manifests as a displacement of fixed points in the world coordinate system in the pixel coordinate system. Three coordinate transformations are required for the transformation from the world coordinate system to the pixel coordinate system, and these three transformations represent the camera’s imaging process. Firstly, the world coordinate system remains unchanged, and in preliminary experiments, the position of the calibration board did not change. Secondly, the camera remains stationary. However, during the thermal transfer process of the camera, the camera’s outer casing and lens are also affected by temperature, resulting in slight deformations. In a static environment, we can assume that the camera coordinate system remains unchanged.

It is worth noting that during operation, the imaging sensor of the camera is the largest heat source, consisting of N × N photosensitive elements arranged. The physical size of the photosensitive elements is on the micron scale. Thermal effects such as the expansion, displacement, and rotation of the photosensitive elements cause changes in the image coordinate system relative to the camera coordinate system, resulting in pixel drift. Additionally, the transformation from the image coordinate system to the pixel coordinate system involves only translation and unit conversion, with no relative changes between them.

**Figure 5:**
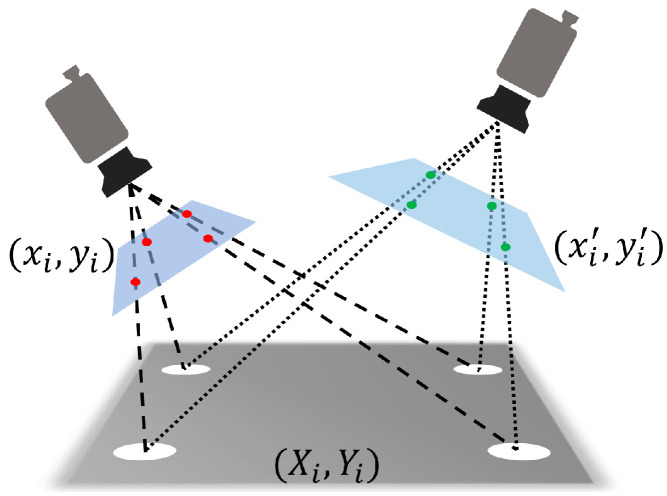
The illustration of perspective transformation.

Based on the above analysis, the fundamental cause of thermal-induced camera error lies in the changes in the imaging sensor relative to the camera coordinate system, leading to the displacement of static targets in the pixel coordinates. The thermal effects on the imaging sensor include expansion, contraction, translation, and spatial rotation, with almost no shear deformation occurring. We only need to find the perspective transformation relationship between the imaging sensor before and after the change to compensate for the thermal-induced pixel drift. As shown in Figure 5, given that the pixel coordinates of the red and green points in their respective images are ($x_{i}$, $y_{i}$) and (${x^{\prime }}_{i}$, ${y^{\prime }}_{i}$), respectively, we can solve for the perspective transformation matrix *M* (perspective transform matrix). Using the perspective transformation matrix, we can convert the coordinates of points between the two surfaces. The perspective transformation process between corresponding points in two surfaces is represented as follows:

(1) $$\left[\begin{array}{c} x_{i}\\ y_{i}\\ 1 \end{array}\right]=\left[\begin{array}{ccc} h_{11} & h_{12} & h_{13}\\ h_{21} & h_{22} & h_{23}\\ h_{31} & h_{32} & 1 \end{array}\right]\left[\begin{array}{c} {x^{\prime }}_{i}\\ {y^{\prime }}_{i}\\ 1 \end{array}\right]$$

$h_{11}$, $h_{12}$, $h_{13}$, $h_{21}$, $h_{22}$, $h_{23}$, $h_{31}$, and $h_{32}$ are elements of the perspective transformation matrix *M*. The coordinates of each corresponding pair of points can be used to construct two linear equations based on the form of the perspective transformation matrix and then solve for the two matrix parameters. The perspective transformation matrix contains eight unknown parameters, so we need at least two sets of points, each consisting of four points, to solve for the perspective transformation matrix.

(2) $$\left\{\begin{array}{c} x_{i}a_{11}+y_{i}a_{12}+a_{13}-{x^{\prime }}_{i}x_{i}a_{31}-{x^{\prime }}_{i}y_{i}a_{32}-{x^{\prime }}_{i}=0\\ x_{i}a_{21}+y_{i}a_{22}+a_{23}-{y^{\prime }}_{i}x_{i}a_{31}-{y^{\prime }}_{i}y_{i}a_{32}-{y^{\prime }}_{i}=0 \end{array}\right.$$

Next, construct matrix *A* for solving the perspective transformation matrix *M*. In matrix *A*, these equations will be represented in the following form:

(3) $$A=\left[\begin{array}{c} \begin{array}{ccc} \begin{array}{ccc} x_{1} & y_{1} & 1\\ 0 & 0 & 0 \end{array} & \begin{array}{ccc} 0 & 0 & 0\\ x_{1} & y_{1} & 1 \end{array} & \begin{array}{ccc} -{x^{\prime }}_{1}x_{1} & -{x^{\prime }}_{1}y_{1} & -{x^{\prime }}_{1}\\ -{y^{\prime }}_{1}x_{1} & -{y^{\prime }}_{1}y_{1} & -{y^{\prime }}_{1} \end{array}\\ \begin{array}{ccc} x_{2} & y_{2} & 1\\ 0 & 0 & 0 \end{array} & \begin{array}{ccc} 0 & 0 & 0\\ x_{2} & y_{2} & 1 \end{array} & \begin{array}{ccc} -{x^{\prime }}_{2}x_{2} & -{x^{\prime }}_{2}y_{2} & -{x^{\prime }}_{2}\\ -{y^{\prime }}_{2}x_{2} & -{y^{\prime }}_{2}y_{2} & -{y^{\prime }}_{2} \end{array} \end{array}\\ \begin{array}{ccc} \begin{array}{ccc} \vdots & \vdots & \vdots \\ x_{4} & y_{4} & 1\\ 0 & 0 & 0 \end{array} & \begin{array}{ccc} \vdots & \vdots & \vdots \\ 0 & 0 & 0\\ x_{4} & y_{4} & 1 \end{array} & \begin{array}{ccc} \vdots & \vdots & \vdots \\ -{x^{\prime }}_{4}x_{4} & -{x^{\prime }}_{4}y_{4} & -{x^{\prime }}_{4}\\ -{y^{\prime }}_{4}x_{4} & -{y^{\prime }}_{4}y_{4} & -{y^{\prime }}_{4} \end{array} \end{array} \end{array}\right]$$

Further, construct the system of linear equations:

(4) A·h=0

Here, A is the matrix constructed from the previous step, and h is the column vector containing all the unknowns of the perspective transformation matrix *M*.

In practical applications, the collected data often contain some noise errors, resulting in no real solutions for the linear system of equations. During data collection, it is common to increase the number of data sets so that the number of linear equations constructed is greater than the number of unknowns. Then, singular value decomposition (SVD) is used to find the least squares solution to the linear system of equations. The matrix A is decomposed using singular value decomposition, denoted as follows:

(5) $$A=U\Sigma V^{T}$$

(6) $$A=\left[\begin{array}{ccc} u_{11} & \cdots & u_{1m}\\ \vdots & \ddots & \vdots \\ u_{m1} & \cdots & u_{mm} \end{array}\right]\left[\begin{array}{ccccc} \sigma_{1} & 0 & 0 & \ldots & 0\\ 0 & \sigma_{2} & 0 & 0 & 0\\ 0 & 0 & \sigma_{3} & 0 & 0\\ 0 & 0 & 0 & \ddots & 0\\ 0 & 0 & 0 & 0 & \sigma_{n}\\ \vdots & \vdots & \vdots & \vdots & 0\\ 0 & 0 & 0 & 0 & 0 \end{array}\right]{\left[\begin{array}{ccc} v_{11} & \cdots & v_{1n}\\ \vdots & \ddots & \vdots \\ v_{n1} & \cdots & v_{nn} \end{array}\right]}^{T}$$

where

*U* is a square matrix with the same number of rows as *A*, and it is orthogonal.

*Σ* is a diagonal matrix, with its diagonal elements being the singular values of *A*, arranged in descending order. *V^T^* is another square matrix, also orthogonal. The columns of *V* are the right singular vectors of *A*, which form an orthogonal basis for the column space of *A*.

## 3. Thermal-Induced Pixel Drift Phenomena and Compensation Experiments

### 3.1. Pixel Drift Caused by Camera Self-Heating

**Figure 6:**
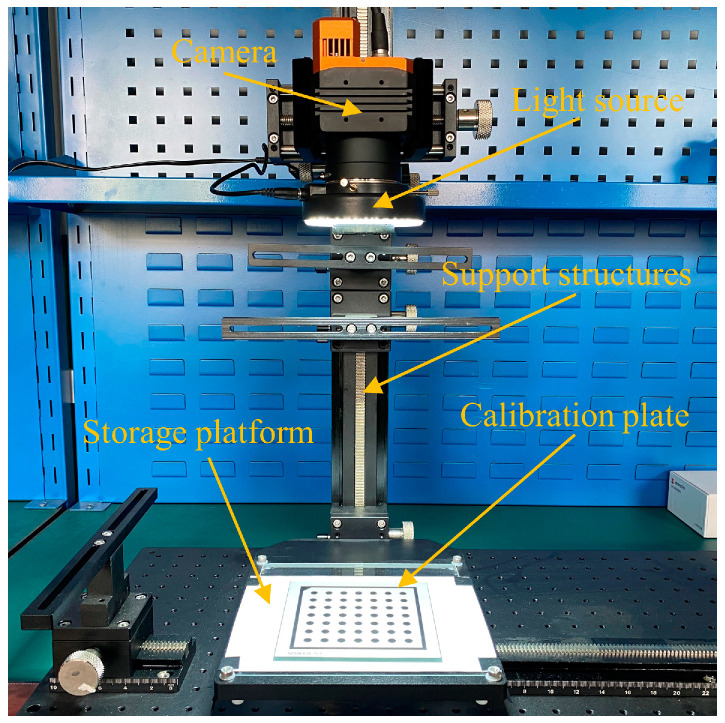
The experimental platform.

To explain the phenomenon of the gradually increasing internal diameter results measured by the deep hole measurement instrument, we set up an experimental platform to test the pixel drift phenomenon of the camera, as shown in Figure 6. The experimental platform mainly includes supporting structures, a camera, light sources, a specimen platform, a calibration board, etc. The camera used is the Hikvision MV-CH250-25TM camera; the lens is the Xenon-Emerald 2.2/50 lens; focal length: 50 mm; max. sensor size: 43.2 mm. A ceramic-based calibration board with an extremely low thermal expansion coefficient of 10^−5^ to 10^−6^/°C was used, which undergoes minimal geometric changes. The ceramic substrate is embossed with a grid of 7 × 7 circles with a diameter of 6.25 mm, spaced 12.5 mm apart. The circle size accuracy of the calibration board is ±0.001 mm.

After opening the camera, image acquisition begins, with one frame captured every 3 s. We employed the Circle Hough Transform [23] to recognize and locate circles in each frame image, outputting pixel coordinates for the centers of 49 circles. The whole collection process lasted 2.5 h, and a total of 3008 sets of data were obtained from this collection, each containing the coordinates of 49 points.

**Figure 7:**
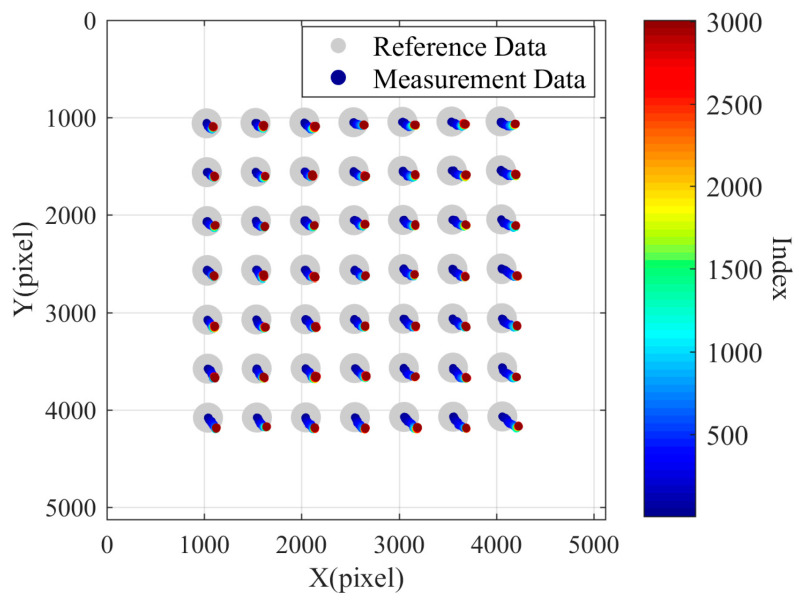
The pixel drift chart of the first set of tests.

Using the first set of data as the reference data, the remaining 3007 sets of data were considered measurement data. The pixel offsets of the centers of the 49 circles in each frame image relative to the first frame image were calculated. As shown in Figure 7, the scatter plot is plotted in the pixel coordinate system, with the larger gray circle indicating the circular spot on the calibration plate in the first frame image. The circles representing measurement data were drawn smaller, and different colors were mapped using the jet color map based on their respective data group index values. It is worth noting that the offset distances of the center points of the measurement data circles relative to the reference data circles were magnified by 100 times. It can be observed that over time, the center points of the circles in the pixel coordinate system underwent significant changes, with their distances from each other increasing while experiencing overall displacement. In this case, the maximum pixel drift distance is 1.99 pixels, the corresponding X-direction drift is 1.704905 pixels, the corresponding Y-direction drift is 1.032532 pixels, the average value of the X-direction pixel drift is 0.977120 pixels, and the average value of the Y-direction pixel drift is 0.690437 pixels. This test result explains the phenomenon of the gradually increasing measured inner diameter observed during instrument measurement.

**Figure 8:**
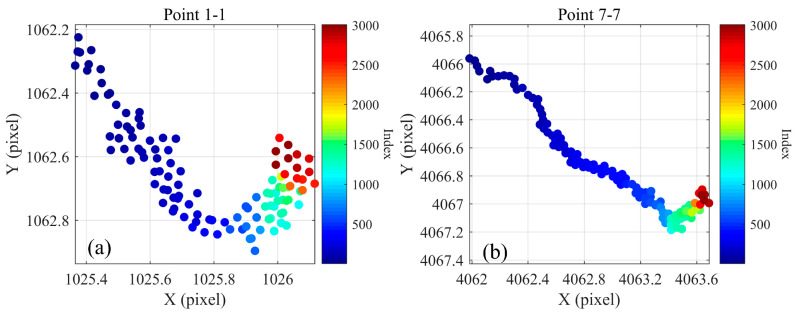
The pixel drift charts: (**a**) pixel drift for point 1-1; (**b**) pixel drift for point 7-7.

We numbered the 49 circular spots on the calibration plate, with the first row of circular spots numbered 1-1, 1-2 ... 1-7, and the seventh row of circular spots denoted 7-1, 7-2...7-7. Figure 8 shows the pixel drift trajectories of points 1-1 (a) and 7-7 (b). It can be observed that point 1-1 moved 0.7 pixels in the X-direction and 0.77 pixels in the Y-direction. The pixel drift ratios here are in true proportion. Point 7-7 moved 1.25 pixels in the X-direction and 1.7 pixels in the Y-direction.

**Figure 9:**
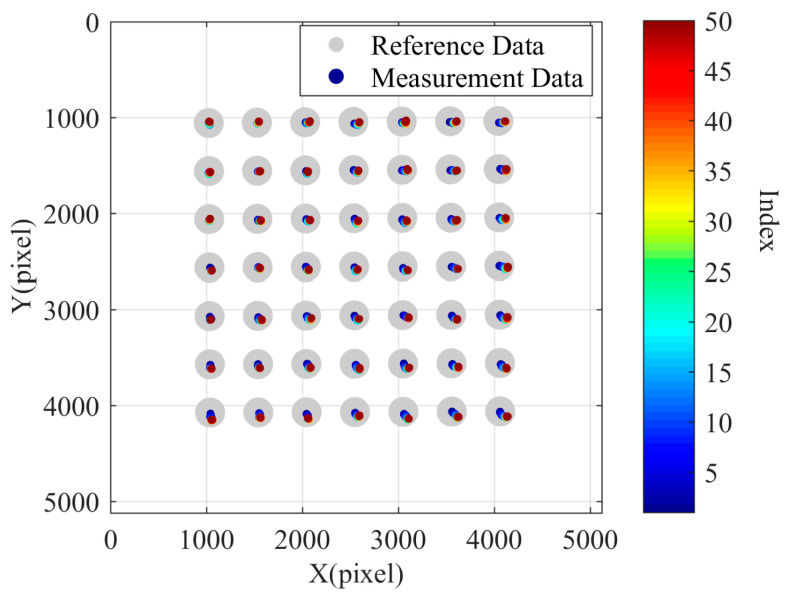
The pixel drift chart of the second set of tests.

To verify that the pixel drift in the camera image acquisition process is caused by temperature, we conducted the second set of data collection. Inside a temperature-controlled chamber, we opened the camera to capture one frame, immediately closed it, waited for 4 min, then reopened the camera, and captured another frame. The experiment lasted approximately 3.4 h, during which we captured a total of 51 frames. We recorded the indoor temperature every 5 min, ensuring that the temperature variation remained within 0.5 degrees Celsius during image acquisition. Using the first frame as the reference image, we plotted the pixel drift, as shown in Figure 9. We found that pixel drift still existed, but the drift magnitude was reduced. It is important to note that the offset distance of the measured data points relative to the reference data points in the figure is magnified by 100 times.

After analyzing the reasons, we speculated that during the 4 min interval between captures, the temperature of the CCD sensor did not return to its initial level, resulting in the cumulative heating of the camera’s imaging sensor and causing pixel drift. Since there was no method to monitor the temperature of the CCD surface, we increased the capture interval to 15 min and conducted the third set of data collection.

**Figure 10:**
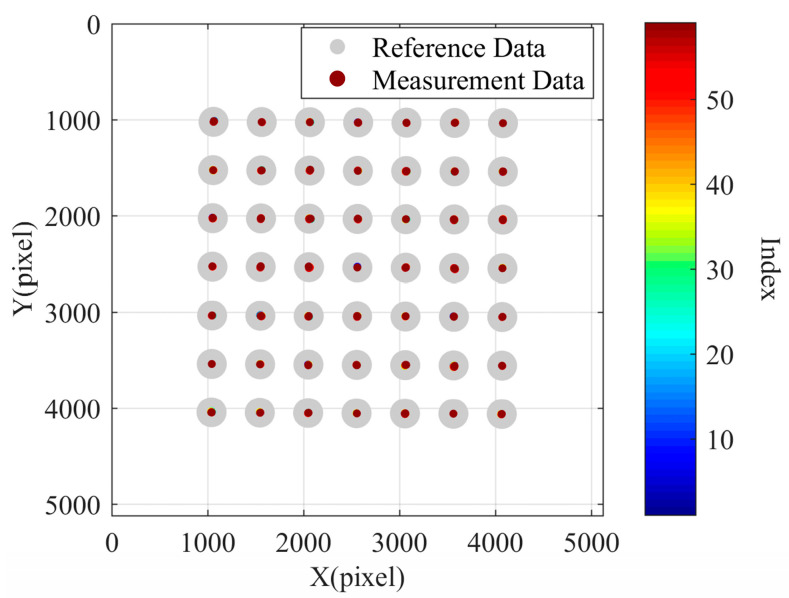
The pixel drift chart of the third set of tests.

The third set of data collection was also conducted in the temperature-controlled chamber. The entire process lasted approximately 15 h, during which we collected a total of 61 frames. Based on the captured images, we plotted the pixel drift, as shown in Figure 10, with the coordinate deviation values magnified by 100 times. It can be observed that the data points in the figure are mostly aligned with the reference data points. This indicates that the heating of the camera’s CCD plane indeed caused pixel drift, which persisted throughout the image acquisition process and affected the measurement results.

### 3.2. The Validation of the Compensation Method

**Figure 11:**
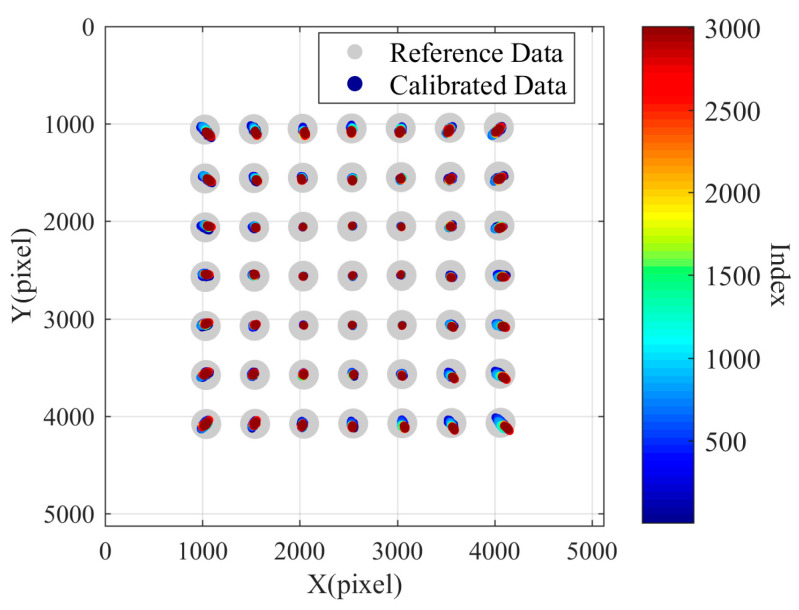
Compensation results calculated with nine points in the central area.

In the initial design phase, to fully utilize the imaging surface of the camera and improve the effective pixels of the laser ring image, the laser ring is imaged at the edge of the image, with the target set in the middle area of the image. That is, during compensation, we use the coordinate changes of the target in the middle area to predict the pixel drift in the peripheral areas of the image. Using the first set of data collected in Experiment 3, with the first frame image as the reference image and the measurement results of the first frame image as the reference results, nine points in the middle of each set of measurement data are selected as feature points. Starting from the second set of measurement data, the perspective transformation matrix *M*(i) between the coordinates of the feature points in the i-th frame image and the coordinates of the feature points in the first frame image is calculated separately. The perspective transformation matrix describes the transformation process from the i-th set of feature point data to the first set of feature point data. Then, all the points in the 3007 sets of data are transformed using the corresponding perspective transformation matrix to obtain 3007 sets of transformed data. Similarly, taking the first set of data points as reference data, the remaining 3007 sets of transformed data are taken as calibrated data. A scatter plot is drawn in the coordinate system, as shown in the Figure 11. It is important to note that the offset distance of the centroid of the calibration data relative to the centroid of the reference data is magnified by 100 times in the graph. In the figure, it can be seen that the nine points on the inner side of the coordinate area are effectively compensated through perspective transformation matrix conversion, but they fail to effectively predict the pixel drift in the peripheral area.

**Figure 12:**
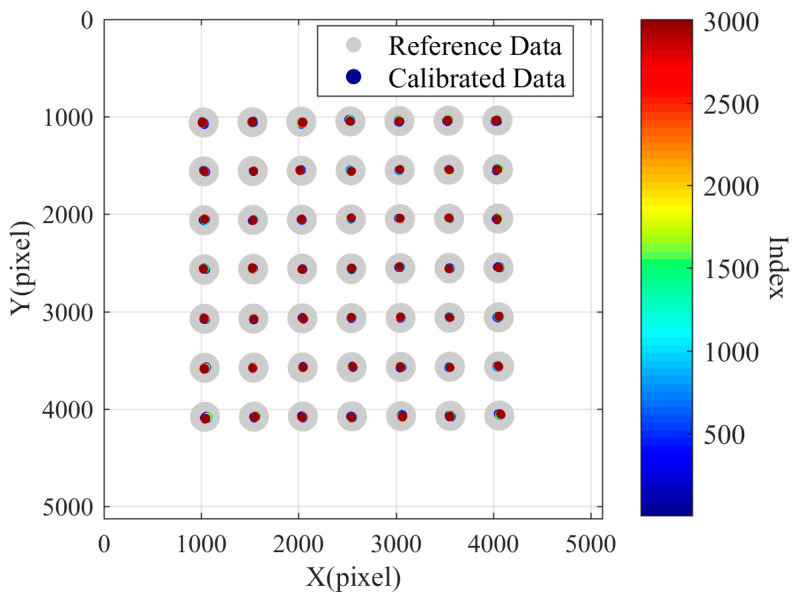
Compensation results calculated with sixteen points in the central circular area.

The perspective transformation matrix is calculated using the 16 points in the middle ring as feature points, and the perspective transformation matrix is applied to compensate all the points to obtain the compensated image as shown in Figure 12. Both inner and outer sides of the 16 points are well compensated. Additionally, the predicted compensation results of the inner points are better than those of the outer points.

**Figure 13:**
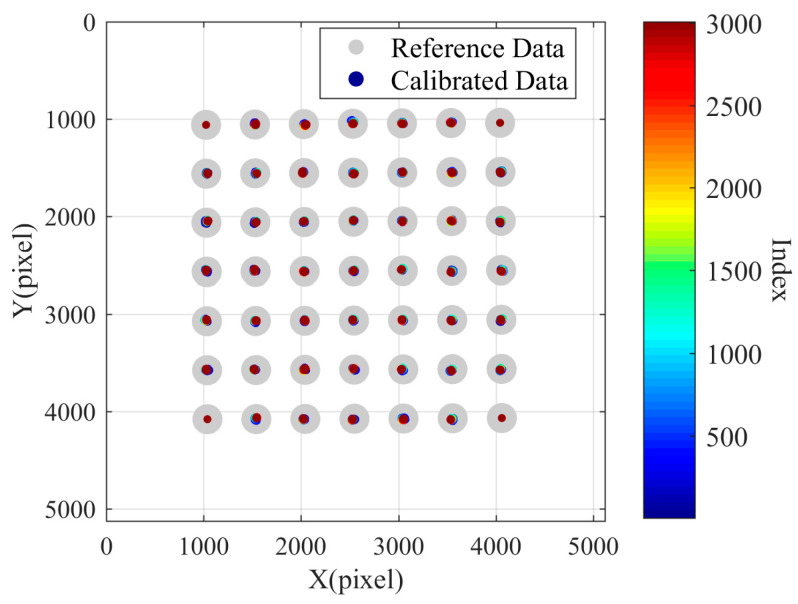
Compensation results calculated using the outer four points.

Using the outermost four points as feature points for compensation, the results are shown in Figure 13. It can be observed that employing the outermost four points as feature points to calculate the perspective transformation matrix for data compensation can effectively correct the thermal pixel drift of the camera.

**Figure 14:**
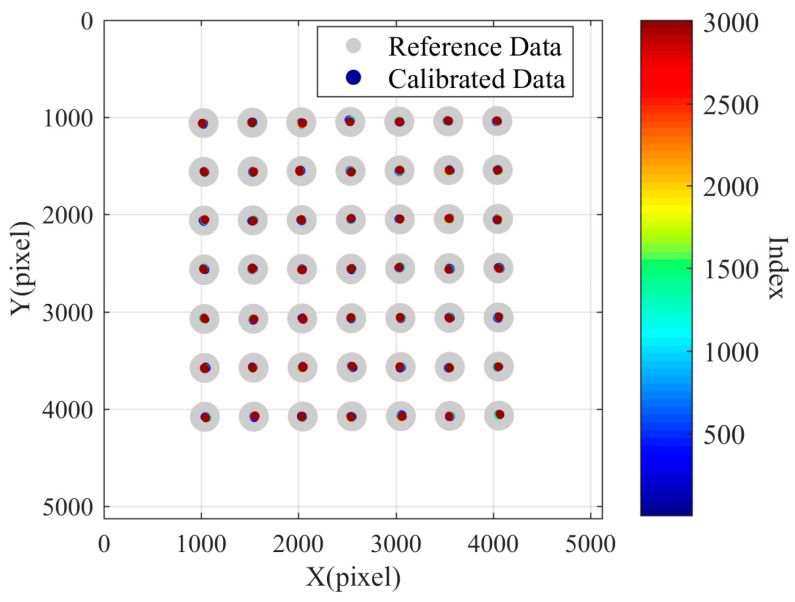
The compensation results with all points involved in the calculation.

The results of applying perspective transformation matrix to all points and performing transformation compensation are shown in Figure 14, with no significant difference observed compared to the compensation results using only the outer four points as features. This indicates that the number of points involved in solving the perspective transformation matrix has a minimal impact on the quality of the matrix solution, whereas the position and distribution of the points involved in the calculation determine the quality of the solution. The perspective transformation matrix transformation can be effectively applied to predict and compensate for pixel drift from the outer to the inner regions of the image.

Specific metrics such as Max offset, Max X-axis offset, Max Y-axis offset, Average X-axis offset, Average Y-axis offset, and time were extracted from the outcomes of different methods for comparison, as shown in Table 1.

**Figure 15:**
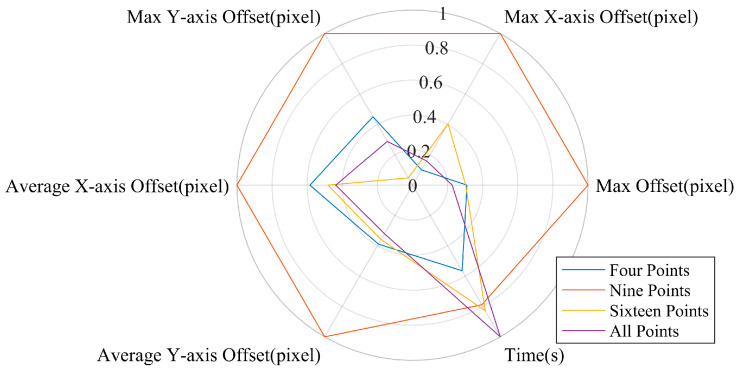
Normalized property map for different methods.

After normalizing these metrics, we plotted the normalized attribute graphs for different methods, as shown in the Figure 15. The advantages of different methods can clearly be seen. The method that uses only the four outer points as feature points for calculation has the shortest processing time. Although it slightly underperforms in Max offset, Max Y-axis offset, Average X-axis offset, and Average Y-axis offset compared to other methods, it has the smallest area in the normalized attribute graph, making it the optimal solution. Using attribute graphs for comparison increases the credibility of the results.

**Figure 16:**
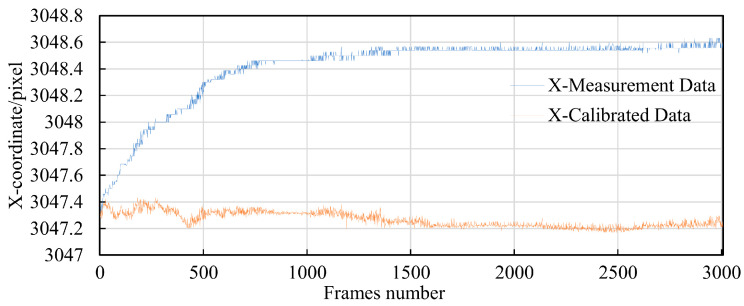
Comparison of X-axis pixel coordinate compensation.

**Figure 17:**
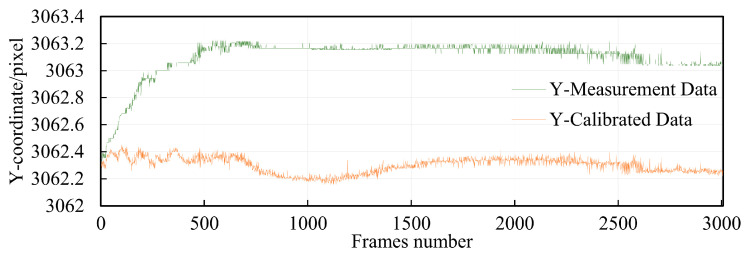
Comparison of Y-axis pixel coordinate compensation.

Randomly selecting point 5-5 as an example for demonstrating the calibration effect, the X and Y parameter curves before and after compensation of point 5-5 are plotted. From Figure 16, it can be observed that the range of pixel offset after X-axis compensation is reduced from 1.3 pixels to 0.264 pixels. and Figure 17 describes that the range of pixel offset after Y-axis compensation is reduced from 0.88 pixels to 0.29 pixels. In probability theory and statistics, the coefficient of variation, also known as the relative standard deviation, is a normalized measure of the dispersion of a probability distribution. The coefficient of variation is defined as the ratio of the standard deviation to the mean, reflecting the dispersion on a unit mean scale. It is commonly used in comparing the dispersion of two populations with unequal means. The coefficient of variation before compensation in the X and Y directions is 0.0000857 and 0.0000467, respectively. After compensation, the coefficients of variation are 0.0000183 and 0.0000186, respectively.

## 4. Repeatability Measurement Test of Deep Hole Measuring Instrument

**Figure 18:**
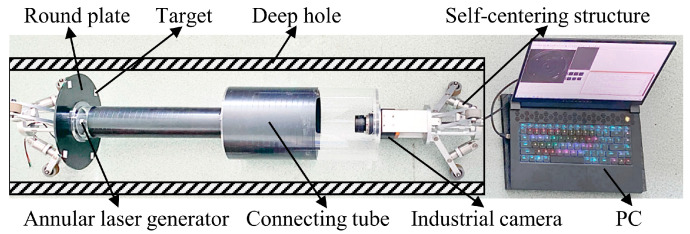
The deep hole measurement instrument with added targets.

In the design of the deep hole inspection instrument, it is advisable to place the targets around the periphery of the camera’s field of view rather than in the central area of the image. Although it is preferable to have as many points as possible involved in the calculation of the perspective transformation matrix, the computation becomes more extensive and time-consuming. In the experimental results, using the four outer points for calculating the perspective transformation matrix has already achieved satisfactory compensation results. As shown in Figure 18, we added a piece of a round plate near the ring laser. Four targets were placed on the round plate, ensuring that they would not obstruct the imaging of the laser ring. When the camera captures images of the ring laser, it can simultaneously capture images of the targets. Since the targets are fixed to the camera, their positions in the camera coordinate system do not change.

In the captured images, the displacement of the targets, changes in the distance between target points, and the rotation of the targets all reflect the camera’s thermal drift. For each image captured by the camera, a perspective transformation matrix can be calculated based on the pixel changes in the targets. By using this perspective transformation matrix to transform the coordinates of the laser ring edge in the current image, compensation for the camera’s thermal error can be achieved.

**Figure 19:**
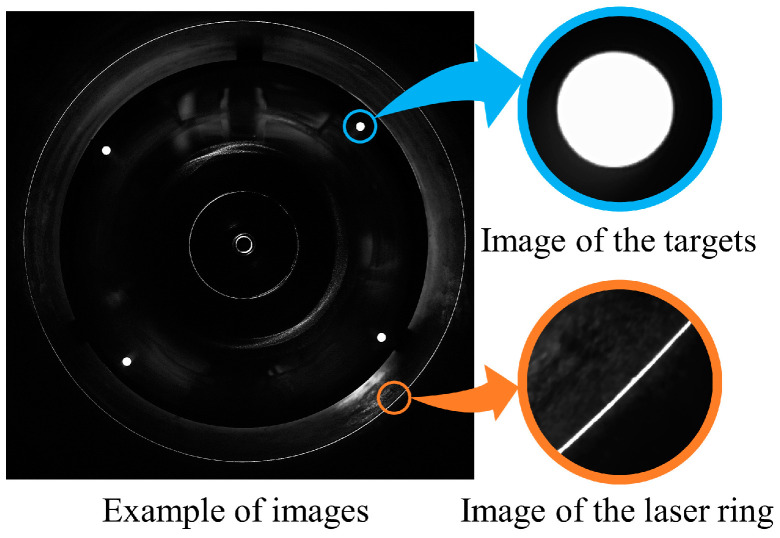
Examples of images captured by the instrument.

The images captured by the measuring instrument are shown in Figure 19. Due to insufficient lighting inside the deep hole, we set up a round plate as hollow black occluders resembling masks, with four circular areas left transparent in the middle as targets. When external light enters the hole, the targets can be imaged well, which is beneficial for subsequent image processing.

**Figure 20:**
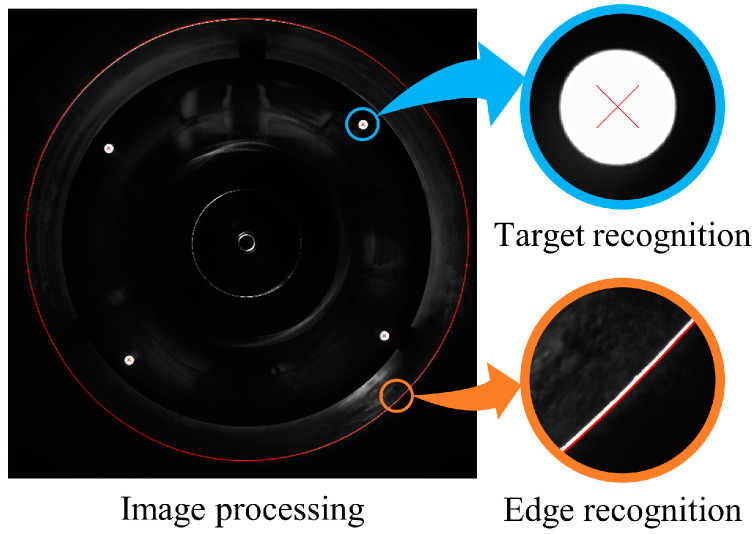
Illustration of image processing.

Figure 20 shows the recognition and extraction results of the target center and the edge of the laser ring. After filtering, enhancing, and segmenting the images, we used the Hough Transform algorithm to identify the target center. The edge recognition and sub-pixel contour extraction of the laser ring were accomplished using the canny operator [24].

**Figure 21:**
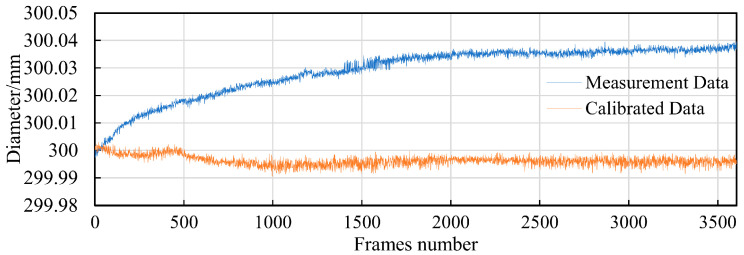
Compensation results of diameter error.

In the repeatability test of the deep hole measuring instrument, we captured one image every 2 s, totaling 3605 frames. The entire testing process lasted for two hours without controlling the ambient temperature. Figure 21 presents the results of the repeatability test of the measuring instrument. It can be observed that using this method, the maximum error in the diameter data measured by the instrument decreased from 0.04 mm to 0.012 mm, effectively compensating for the camera’s thermal error and improving the measurement accuracy of the measuring instrument.

## 5. Discussion and Conclusions

Compared with existing methods for compensating camera-induced thermal errors, the method described in this paper has several advantages [17,18,19,20]. First, it eliminates the need for camera preheating, allowing for measurements to be conducted immediately. This is a significant advantage, as existing compensation methods require the camera to be preheated for an hour or even longer to reach thermal equilibrium before it can be used. Second, the target set can be imaged within the camera, allowing for the real-time monitoring of the actual pixel drift in each frame, thereby facilitating data calibration. This method does not require the consideration of ambient temperature, enabling it to be used in the field or in environments with large temperature variations. Additionally, the system only adds a target board, which does not obstruct the camera’s measurement field of view, introduces no additional error factors, is low in cost, flexible in installation, and easy to deploy.

In this study, we analyzed the causes of camera thermal errors and the compensation theory, proposing a method using perspective transformation to compensate for thermal errors in deep hole measuring instruments. Several targets were set within the camera’s field of view to monitor pixel drift and to solve for the perspective transformation matrix between each frame and the first frame. This matrix is used to predict the pixel drift for each pixel coordinate point within the entire field of view, thereby compensating for thermal-induced pixel drift. The analysis results show that the number of targets involved in the calculation had little effect on the prediction results, while the good distribution of targets in the field of view could yield better prediction results.

To verify the practicality of the compensation method, we conducted repeatability measurement tests on the deep hole measuring instrument. The results show that before compensation, the repeatability measurement error of the instrument was 0.04 mm. After compensation, the measurement error was suppressed to 0.012 mm. Using perspective transformation to compensate for thermal errors in deep hole measuring instruments effectively enhances the repeatability of measurements and has high practical value.

## Figures and Tables

**Table 1:** Comparison of results of different methods.

Category	Nine Points	Sixteen Points	All Points	Four Points
Max offset/pixel	1.271	0.385	0.287	0.393
Max X-offset/pixel	0.947	0.383	0.151	0.095
Max Y-offset/pixel	0.848	0.041	0.244	0.382
Average X-offset/pixel	0.129	0.062	0.057	0.075
Average Y-offset/pixel	0.171	0.062	0.055	0.066
Time/s	3.723	3.913	4.720	2.664

## Data Availability

Data are contained within the article.
